# Inflammatory Cytokines, Immune Cells, and Organ Interactions in Heart Failure

**DOI:** 10.3389/fphys.2021.695047

**Published:** 2021-07-01

**Authors:** Huihui Li, Chen Chen, Dao Wen Wang

**Affiliations:** ^1^Division of Cardiology, Department of Internal Medicine, Tongji Hospital, Tongji Medical College, Huazhong University of Science and Technology, Wuhan, China; ^2^Hubei Key Laboratory of Genetics and Molecular Mechanisms of Cardiological Disorders, Tongji Hospital, Tongji Medical College, Huazhong University of Science and Technology, Wuhan, China

**Keywords:** inflammation, heart failure, cytokine, immune cells, organs

## Abstract

Despite mounting evidence demonstrating the significance of inflammation in the pathophysiological mechanisms of heart failure (HF), most large clinical trials that target the inflammatory responses in HF yielded neutral or even worsening outcomes. Further in-depth understanding about the roles of inflammation in the pathogenesis of HF is eagerly needed. This review summarizes cytokines, cardiac infiltrating immune cells, and extracardiac organs that orchestrate the complex inflammatory responses in HF and highlights emerging therapeutic targets.

## Introduction

Heart failure (HF) is a clinical syndrome characterized by symptoms and signs induced by the structural and/or functional compromise of the heart, presents as dyspnea, fatigue, and fluid retention, and so on (Ponikowski et al., 2016; Yancy et al., 2017). HF is the principal cause of mortality and disability worldwide. In developed countries, the prevalence of HF is 1.5–2.2% (Mosterd and Hoes, 2007). The 1-year all-cause mortality rate of HF patients is 17 and 19.2% in Europe and Asia, respectively (Maggioni et al., 2013; Tromp et al., 2018a). HF could be divided into HF with reduced ejection fraction (HFrEF), HF with midrange ejection fraction, and HF with preserved ejection fraction (HFpEF) (Ponikowski et al., 2016). It is a syndrome caused by the complicated interaction of myocardial damage, neurohormonal activation, inflammatory response, and renal dysfunction (Mann, 2002; Zimmet and Hare, 2006; Braunwald, 2008). Although the etiology and pathogenesis of HF are still perplexing, the persistent inflammation of myocardium is believed to participate in the pathogenesis across the spectrum of HF subtypes in different ways (Dick and Epelman, 2016). Two recent biomarker profiles analyses, Counseling in Heart Failure (COACH) and Biology Study to Tailored Treatment in Chronic Heart Failure (BIOSTAT-CHF) trials, demonstrated a prominent correlation between inflammation and HFpEF, whereas HFrEF was more related to stretch-mediated interactions (Tromp et al., 2017, 2018b). This might partially be explained by the non-cardiac comorbidities of HFpEF patients such as diabetes mellitus, hypertension, obesity, anemia, chronic obstructive pulmonary disease, and chronic kidney disease. All of them are prone to systemic inflammatory state (Paulus and Tschope, 2013). However, most clinical trials using anti-inflammatory agents have provided disappointing results, reflecting the inadequate understanding about the sophisticated inflammatory network within the heterogenous performance of HF. Thus, a better illustration about how specific inflammatory cytokines, immune cells, and extracardiac organs collaborate and influence cardiac function may provide experimental basis for disease intervention and drug discovery.

## Inflammatory Cytokines and Their Roles in HF

Since 1990, levels of several inflammatory mediators, including tumor necrosis factor α (TNF-α), interleukin 6 (IL-6), IL-1β, IL-18, and immunological antigens, were validated to be increased in the plasma of HF patients (Torre-Amione et al., 1996; Anker and von Haehling, 2004; Braunwald, 2008). These suggested the “cytokine hypothesis” that inflammation contributed to the pathogenesis of HF (Seta et al., 1996; Mann, 2015). The elevated circulating cytokines might be produced by cardiac structural cells [cardiomyocytes (Kapadia et al., 1995), endothelial cells (Liu Y. et al., 2014), and fibroblasts (Sandanger et al., 2013)], various cardiac infiltrating immune cells (Pinto et al., 2016), and extracardiac tissues (hypoperfused skeletal muscle, lymphoid organs, intestinal tissue, and adipose tissue) (Murphy et al., 2020). Increased circulating cytokines do not only correlate with the severity of HF, but also possess prognostic value (Rauchhaus et al., 2000; Braunwald, 2008). Up to now, various functions of cytokines in HF were revealed (Table 1).

**TABLE 1:** Cytokines in heart failure.

Cytokines	Functions
TNF-α^✸ ✩◆ □^	Induce inflammatory genes expression and apoptosis, release proinflammatory cytokines, promote adverse remodeling
Fas^✸ ✩◆^	Trigger programmed cell death
IL-1^✸ ✩◆ □^	Induce negative inotropic effect through impairing β-adrenergic responsiveness and disturbing calcium handling
ST2^✸ ✩◆⊚^	Negatively modulate TLR signaling, inhibit nuclear factor κB activation
IL-6^✸ ✩◆ □^	Pleiotropic proinflammatory responses
IL-10^✸ ◆^	Inhibits proinflammatory cytokines secretion, block ROS release, modulate TNF-α-mediated responses

*^✸^Participate in pathogenesis. ^✩^Prognostic value. ^◆^Identify high-risk patients. ^□^Potential therapeutic target. ^⊚^Diagnostic value. TNF-α, tumor necrosis factor-α; Fas, factor associated suicide; IL-1, interleukin 1; ST2, suppression of tumorigenicity 2; TLR, toll like receptor; IL-6, interleukin 6; IL-10, interleukin 10; ROS, reactive oxygen species.*

### Tumor Necrosis Factor

Tumor necrosis factor α is the first cytokine discovered to be elevated in the peripheral blood of HF patients. Increased serum TNF-α level suggested impaired cardiac function and poor prognosis (Torre-Amione et al., 1996; Rauchhaus et al., 2000). TNF-α exerts its function through binding to the receptors. It is toxic with TNF receptor 1 (TNFR1) while protective with TNFR2 (Monden et al., 2007). Constantly increased TNF could attenuate β1-adrenergic responsiveness, induce cell apoptosis, and destroy the balance between matrix metalloproteinases (MMPs) and tissue inhibitor of metalloproteinase, resulting in ventricular hypertrophy, dilatation, and diminished ejection fraction (Kubota et al., 1997; Tang et al., 2004; Zhang et al., 2011). Mice with TNF-α overexpression spontaneously progressed into end-stage dilated cardiomyopathy (DCM) (Tang et al., 2004). However, clinical trials that target TNF-α with infliximab [Anti-TNF Therapy Against Congestive Heart Failure Trial (ATTACH)] or etanercept [Randomized Etanercept Worldwide Evaluation Trial (RENEWAL)] yielded disappointing results and were terminated prematurely due to poor survival improvement and enhanced risk of hospitalization (Chung et al., 2003; Mann et al., 2004). Thus, further studies are needed to better understand the effects of TNF-α in HF.

Factor associated suicide (Fas), also named as APO-1, is a member of TNF receptor family, which is expressed in various tissues and cells including cardiomyocytes. Circulating level of Fas was elevated in HF patients and was associated with the severity of cardiac dysfunction (Okuyama et al., 1997). Blockade of the interaction between Fas and its ligands could improve survival rate and reduce ventricular remodeling in mice with myocardial infarction (MI). Thus, it might be a potential therapeutic target against chronic HF after MI (Li et al., 2004).

### IL-1 Family

It was reported that IL-1 had a close association with HF. In patients with sepsis, IL-1 was considered to be a “soluble myocardial depressant factor” (Van Tassell et al., 2013b). Chronic hypoxia could induce IL-1 production in the cardiomyocytes (Kacimi et al., 1997). Circulating levels of IL-1β and IL-1 receptor antagonist (IL-1Ra) were increased in congestive HF patients (Testa et al., 1996). IL-1 could induce reversible negative inotropic effects on cardiomyocytes both *in vitro* (Liu and Schreur, 1995) and *in vivo* (Van Tassell et al., 2013a) through impairing β-adrenergic responsiveness and calcium handling (Buckley and Abbate, 2018). Meanwhile, blockade of IL-1 could restore calcium homeostasis, reduce inflammatory infiltration, and improve cardiac dysfunction (Van Tassell et al., 2012; Francis et al., 2014; Sager et al., 2015). Previous case reports indicated that in myocarditis-associated end-stage HF patients, blocking IL-1 improved cardiac contractility within 24 h (Cavalli et al., 2016, 2017). The prespecified subanalysis of the Canakinumab Anti-inflammatory Thrombosis Outcome Study (CANTOS) showed that for patients with prior MI and elevated high-sensitivity C-reactive protein (hs-CRP), canakinumab effectively reduced HF-related hospitalization and all-cause mortality at a dose-dependent manner (Everett et al., 2019). Thus, IL-1-targeted therapy may be beneficial to HF patients. Moreover, stratified analysis using multiple indexes revealed that HF patients with higher CRP could benefit more from anti-inflammatory therapy.

Suppression of tumorigenicity 2 (ST2), a decoy receptor of IL-33, is another member of IL-1 cytokine superfamily, which could be secreted by cardiomyocytes under mechanical strain (Weinberg et al., 2003). The increase in soluble ST2 (sST2) was independently and positively associated with poor outcomes in HF patients and might be valuable to predict prognosis (Anand et al., 2014).

### Interleukin 6

Interleukin 6 acts as a downstream of IL-1, which attracted particular attention as a central factor in the pathophysiological processes of several inflammatory conditions. Preclinical researches indicated that IL-6 had pleiotropic adverse effects on cardiovascular system. Increased circulating IL-6 was reported in congestive HF patients, which had a significantly positive correlation with worsening cardiac symptom and renal dysfunction (Deswal et al., 2001; Plenz et al., 2001; Hanberg et al., 2018). Stimulation of isolated cardiomyocytes with IL-6 and soluble IL-6R could induce hypertrophy (Hirota et al., 1995), whereas IL-6 inhibition reduced cardiac hypertrophy and fibrosis in angiotensin II-treated mice (Coles et al., 2007). Although many IL-6-targeted therapies were used in the treatment of rheumatologic diseases and immune checkpoint inhibitor-induced cytokine release syndrome (Kang et al., 2019), clinical trials especially aimed at the therapeutic effects of anti-IL-6 on HF have not been implemented. A recent observational study containing 2,329 patients in the BIOSTAT-CHF cohort demonstrated that increased plasma IL-6 concentration was positively correlated with atrial fibrillation, disturbed iron metabolism, poorer exercise tolerance, higher N-terminal pro-brain natriuretic peptide (NT-proBNP) concentrations, and lower estimated glomerular filtration rate (Markousis-Mavrogenis et al., 2019). Besides, circulating IL-6 concentration was independently predictive of all-cause and cause-specific mortality (Markousis-Mavrogenis et al., 2019).

### Interleukin 10

Interleukin-10 is generally considered as an anti-inflammatory cytokine with pleiotropic function. It inhibits the secretion of various proinflammatory cytokines, especially TNF-α (Kaur et al., 2006; Ouyang et al., 2011). The protein and mRNA levels of membrane-bound IL-10 were dramatically reduced in the heart of mice with acute MI (AMI) (Kaur et al., 2006). Moreover, it was negatively correlated with the cardiac function and progression to congestive HF (Kaur et al., 2006). In the heart samples of DCM patients, IL-10 expression was decreased and negatively associated with the disease severity (Ukimura et al., 2003). Although there has yet to be a clinical trial that specifically regulates IL-10 in HF patients, treatment with growth hormone or intravenous immunoglobulin has been shown to be associated with a marked increase in plasma IL-10 level and consequently improved cardiac contractile performance in HF patients (Gullestad et al., 2001; Adamopoulos, 2003).

## Immune Cells and Their Roles in HF

The heart harbors all of the major immune cell types in the steady state, including monocytes, macrophages, T cells, neutrophils, B cells, dendritic cells (DCs), natural killer (NK) cells, and mast cells (Pinto et al., 2016). The number of immune cells in the heart from a healthy adult mouse is more than 10-fold than in skeletal muscle (Ramos et al., 2017). Immune cells participate in the pathogenesis of various inflammatory and non-inflammatory cardiovascular diseases (Adamo et al., 2020). Previous researches revealed a positive correlation between peripheral blood immune cell level and left ventricular dysfunction both in animal model and HF patients (Yndestad, 2003; Fukunaga et al., 2007a; Pistulli et al., 2016). Apart from circulating inflammatory cells, transcriptional sequencing of human heart samples showed diverse expression profiles of innate immune responses related genes between failing and non-failing hearts (Mann et al., 2010). Endomyocardial biopsy in HF and DCM patients revealed a 30% detection rate of myocardial inflammatory infiltration (Kuhl and Schultheiss, 2012). A research using dual-target positron emission tomography (PET)/magnetic resonance imaging to monitor the size of immune cell population indicated a large number of inflammatory monocytes, macrophages, and neutrophils existed in the cardiac tissue after MI (Keliher et al., 2017). Another research demonstrated that inflammatory cells especially macrophages and T cells infiltrated in the heart of DCM patients without any discernible viral infection history (Noutsias et al., 2002). Studies from different animal models have revealed various potential therapeutic targets focusing on these immune cells in HF (Table 2).

**TABLE 2:** Main immune cells in heart failure.

Immune cells	Functions	Potential therapeutic targets from animal studies
Macrophages	Elevated CCR2^+^MHCII^high^Ly6c^+^ macrophages in myocardium could secrete inflammatory cytokines and contribute to ventricular dysfunction	CCR2–CCL2 signaling axis
Mast cells	Attenuate left ventricular remodeling and promote cardiac dysfunction	Mast cell depletion
Neutrophils	Destructive at acute stage and protective at chronic stage	Annexin A1
Natural killer cells	Release cytokines and modulate immune system	
Dendritic cells	Protective at acute stage and destructive at chronic stage	
CD4^+^ T cells	T_*H*_1 cells are mainly proinflammatory	
	T_*H*_2 cells are mainly profibrosis	
	T_*H*_17 cells contribute to cardiac hypertrophy and promote adverse cardiac remodeling	
B cells	Induce direct myocardial injury, produce inflammatory cytokines, and antibodies	B-cell depletion

### Macrophages

Macrophages, one of the most abundant immune cell types in the heart, are commonly divided into M1 and M2 types due to cell surface markers and their functions in inflammatory responses. Recently, this classification criterion was considered imperfect because of the plasticity and highly variable cell surface marker expression of macrophages. Evidence from single-cell sequencing and genetic fate mapping indicated that the expression levels of CCR2 and MHC-II were sufficient to classify macrophage populations in adult mouse heart (Epelman et al., 2014; Hulsmans et al., 2017; Lavine et al., 2018). At healthy status, heart macrophages were dominated by CCR2^–^ macrophages, which could further divide into two categories by MHCII expression (CCR2^–^MHCII^high^ macrophage and CCR2^+^MHCII^low^ macrophage). CCR2^–^ macrophages originate from embryonic precursor, mainly function in coronary system development, angiogenesis, and immune quiescence (Epelman et al., 2014; Lavine et al., 2014). In addition, CCR2^–^MHCII^high^ macrophages have a special role in presenting antigens to T cells (Epelman et al., 2014; Leid et al., 2016). A small, but more proinflammatory CCR2^+^MHCII^high^ macrophage populations also exist in the healthy heart, which were maintained and renewed by circulating Ly6C^high^CCR2^+^ monocytes influx (Leid et al., 2016). In the heart tissue of transverse aortic constriction-induced HF mice, myocardial expression of CCR2 ligand such as CCL2, CCL7, and CCL12 was enhanced, accompanied with significantly increased proinflammatory monocyte-derived CCR2^+^ macrophages (Xia et al., 2009; Liao et al., 2018; Patel et al., 2018). The increased CCR2^+^ macrophages could produce inflammatory cytokines and chemokines, resulting in cardiac T-cell expansion, contributing to cardiomyocyte damage, cardiac remodeling, and pathological hypertrophy (Patel et al., 2018). Clinically, the abundance of CCR2^+^ macrophage was positively associated with adverse left ventricular remodeling and persistent left ventricular dysfunction in HF patients (Bajpai et al., 2018; Dick et al., 2019). Thus far, no CCR2 modulating therapy has been approved for clinical indications. But previous animal experiments targeting the CCR2-CCL2 signaling axis through various approaches such as CCR2 antagonists and monoclonal antibody (Hilgendorf et al., 2014; Liao et al., 2018; Patel et al., 2018), CCR2-targeting PEG-DSPE micelles (Wang et al., 2018), RNA silencing technique targeted on endothelial cell adhesion molecules (Sager et al., 2016), silencing of macrophage polarization factor IRF5 (interferon regulatory factor 5) (Courties et al., 2014), immune-modifying microparticles infusion (Getts et al., 2014), and CCR2-targeted lipid nanoparticle-encapsulated small interfering RNA (Gordon, 2012) had obtained therapeutic benefits both in ischemic and non-ischemic HF by attenuating the proinflammatory monocyte infiltration in the myocardium. These results highlight the potential of CCR2-CCL2 signaling axis-targeted therapy. Apart from that, a recent single-cell sequencing study indicated that in pressure overload HF mice, CCR2^+^M1 like proinflammatory macrophages, expressed a high level of oncostatin M (OSM) (Martini et al., 2019), which exerted a major role of cardiomyocyte dedifferentiation and remodeling during AMI and in DCM (Kubin et al., 2011). OSM was identified to mediate the TNF-α-resistant effect in inflammatory bowel disease patients. This might partially explain the refractivity of HF patients to anti-TNF-α therapy (West et al., 2017).

### Mast Cells

Mast cells were originally defined as effectors of allergy and anaphylactic reactions. However, recent researches have validated that cardiac mast cells also participated in other physiological processes, including vascular homeostasis and angiogenesis (da Silva et al., 2014). Mast cells harbor granules that store histamine, proteases, various cytokines, chemokines, and growth factors in the cytoplasm and exert their function through degranulation (Mukai et al., 2018). Researches indicated that the number of mast cells was significantly increased in the heart of end-stage cardiomyopathy patients, which promoted cardiac adverse remodeling through activating MMPs and myocardial fibrillar collagen degradation (Akgul et al., 2004; Levick et al., 2011). Either depletion of mast cells or inhibition of their degranulation could attenuate left ventricular remodeling and cardiac dysfunction, as well as improve survival rate in animal models (Hara et al., 2002; Brower and Janicki, 2005; Liu Y. H. et al., 2014). Thus, cardiac mast cell population may be a potential target for cardioprotection.

### Neutrophils

Neutrophils are the most abundant type of circulating leucocytes in human, and recognized as the first responder to acute inflammatory response. Cumulative evidence indicated that neutrophils played a pivotal role in chronic inflammation as well (Bonaventura et al., 2019). Neutrophils participate in various cardiovascular diseases via releasing degranulation products, recruiting and activating macrophages and pDCs, delivering microvesicle and cytokine, and so on (Bonaventura et al., 2019). Some researches indicated that the blood count of neutrophil was positively correlated with the severity of coronary damage in coronary artery disease patients (Sharma et al., 2017). Neutrophil/lymphocyte ratio could predict acute HF patients with a higher risk of vascular events (Uthamalingam et al., 2011). In patients with AMI that developed congestive HF, 92.5% had relative neutrophilia (neutrophil percentage >65%), whereas in patients with AMI that did not develop into congestive HF, the incidence of neutrophilia was 45% (Kyne et al., 2000). Previously, it was believed that neutrophils exerted a proinflammatory effect and augmented heart damage in MI. The increased counts or volume of circulating neutrophils after MI was positively correlated with infarction size and negatively correlated with left ventricular function and clinical outcomes (Chia et al., 2009; van Hout et al., 2015). However, recently, researchers found that in infarct healing process, neutrophils could promote macrophages polarization toward a proreparative and proangiogenesis phenotype through releasing gelatinase-associated lipocalin (NGAL) and annexin A1 (Horckmans et al., 2017; Ferraro et al., 2019). Accordingly, depletion of neutrophils in mice led to worsening cardiac function, increased cardiac fibrosis, and enhanced expression of HF biomarkers after MI (Horckmans et al., 2017). Besides, annexin A1 knockout mice subjected to MI were stagnated in macrophages repolarization and exhibited impeded healing after MI. Annexin A1 treatment significantly improved cardiac function both in mice and pig (Ferraro et al., 2019). In addition, another research indicated that OSM produced by neutrophils and macrophages after MI could induce the release of regenerating islet-derived protein 3β (Reg3β), an essential regulator of macrophage trafficking, from dedifferentiating cardiomyocytes, which further promoted the accumulation of proreparative macrophages in the damaged heart (Lorchner et al., 2015). Thus, special attention should be given on the timing of neutrophil targeted therapy in respect of the clinical course pattern of neutrophil function in HF.

### NK Cells

Natural killer cells were primarily recognized as the major effector lymphocytes of innate immune responses endowed with constitutive cytolytic functions. They play a significant role in repairing damaged tissue and maintaining tissue homeostasis (Tosello-Trampont et al., 2017). In addition, NK cells possess complex biological functions in modulating the immune system through receptor-ligand interactions or release various cytokines and chemokines, such as enhancing the antigen-presenting ability of DCs and dampening macrophage/T-cell responses (Vivier et al., 2011; Ong et al., 2017). Circulating NK cells were dramatically reduced in number with diminished cytolytic function in HF patients, as well as coronary heart disease patients and ischemic heart disease patients (Anderson et al., 1982; Vredevoe et al., 2004; Jonasson et al., 2005; Hou et al., 2012; Backteman et al., 2014). Consistent NK cell deficiency was correlated with low-grade chronic cardiac inflammation, while cardiac inflammation was diminished in patients with restored circulating NK cells (Vredevoe et al., 2004; Backteman et al., 2014). Whether this observation is causative or merely a concomitant phenomenon remains to be clarified. The cytolytic impairment of NK cells was associated with increased IL-6 level, but the underlying molecular mechanism was not revealed (Vredevoe et al., 2004). By preventing inflammatory cell accumulation and limiting collagen production from cardiac fibroblasts, NK cells could suppress the development of cardiac fibrosis (Ong et al., 2017). Further investigation is urgently needed to clarify the role of NK cells in HF.

### Dendritic Cells

As the sentinels of immune system, DCs serve as a bridge linking adaptive and innate immune responses (Mildner and Jung, 2014). Heart-specific self-peptide loaded DCs were capable to induce CD4^+^ T-cell-mediated myocarditis and autoimmune HF in mice (Eriksson et al., 2003). In AMI, the migration and accumulation of DCs to the infarction site were increased and DC depletion resulted in worsening post-MI remodeling (Anzai et al., 2012). The number of proinflammatory monocytes and macrophages increased in the myocardium of DC-depleted mice, indicating that DCs might act as an immune-protective regulator during the postinfarction healing process via regulating monocyte/macrophage homeostasis (Anzai et al., 2012). However, DC infiltration was decreased in the cardiac tissue of symptomatic DCM patients, which indicated a damaged ejection fraction (Pistulli et al., 2013). Thus, the function of DCs may be different in acute and chronic HF.

### T Cells

T cell is the major element of the adaptive immune response. Initial evidence implicated that T cells that participated in the pathogenesis of HF came from the elevated T cell-generated cytokines, IL-2 and IL-10, in the plasma of HF patients (Marriott et al., 1996). Then, circulating T cells from congestive HF patients were validated to have enhanced expression of T-cell activation markers (CD25 and CD69), chemokines, and proinflammatory cytokines [TNF-α, interferon γ (IFN-γ), and IL-18] (Yndestad, 2003). Circulating inflammatory cytokines produced by T cells had a positive correlation with left ventricular dysfunction in chronic ischemic HF and idiopathic DCM patients (Fukunaga et al., 2007b). The proportion of regulatory T (Treg) cells in the plasma of HFrEF patients was decreased with less suppressive activity, whereas the proportion of proinflammatory T-helper 17 (T_*H*_17) cells was increased (Li N. et al., 2010; Tang H. et al., 2010; Tang T. T. et al., 2010; Okamoto et al., 2014). The number of Treg cells was negatively associated with the levels of NT-proBNP, CRP, and IL-6 and possessed a prognostic value in predicting cardiac function (Tang et al., 2011; Okamoto et al., 2014).

CD4^+^ and CD8^+^ T cells, as well as CD4^+^ subsets (T_*H*_1, T_*H*_2, T_*H*_17, and Treg cells), are also infiltrated in the failing heart (Nevers et al., 2015; Martini et al., 2019). Although bulk blockade of CD4^+^ T cells in mice prevented cardiac remodeling and exhibited preserved contractile function (Laroumanie et al., 2014), the roles of CD4^+^ T-cell subtypes are quite different. T_*H*_1 cells are mainly proinflammatory and could activate proinflammatory macrophages, whereas T_*H*_2 responses are mainly profibrotic. T-bet, a T_*H*_1 cell-specific transcription factor, was detected to be elevated in hypertrophic myocardium of patients. T-bet deficiency improved pressure overload-induced cardiac remodeling in rats, indicating the potential therapeutic value of T-bet for HF treatment (Ma et al., 2018). T_*H*_17 cells have been reported to contribute to cardiac hypertrophy and promote adverse cardiac remodeling (Frieler and Mortensen, 2015). IL-17 is an effector molecule of T_*H*_17 cells; blockade of IL-17 cells was beneficial in DCM and MI disease models (Baldeviano et al., 2010; Liao et al., 2012). Treg cells are known to negatively regulate the immune response and suppress the effector functions of T-helper cells (Meng et al., 2016). But a recent research indicated that in non-reperfused MI-induced HF mice, myocardial infiltrating CD4^+^Foxp3^+^ Treg cells exhibited proinflammatory T_*H*_1-type features with the expression of IFN-γ, TNF-α, and TNFR1 and had decreased immunomodulatory capacity with potentiated antiangiogenic and profibrotic properties. Periodic Treg-cell depletion reversed left ventricular remodeling, reduced cardiac fibrosis, and improved neovascularization. Treg cell reconstitution after their depletion could restore immunomodulatory capacity (Bansal et al., 2019). Besides, in mice with existing left ventricular failure, administration of IL-2 (also named as T-cell growth factor) significantly increased Tregs in the lung, consecutively reduced pulmonary macrophages and CD8^+^ T cell infiltration, and attenuated right ventricular hypertrophy (Wang et al., 2016). Thus, Treg cells may be a potential high-yield target for the treatment of ischemic cardiomyopathy and HF. Furthermore, single-cell sequencing of the heart tissues from pressure overload HF mice indicated that Treg-cell population expressed a high level of programmed cell death protein 1 (PD-1), which might partially explain the cardiac toxicity during anti-PD-1 cancer immunotherapy (Martini et al., 2019).

### B Cells

The density of B-cell population increased in the myocardium of both acute cardiac MI and pressure-overload HF mice (Yan et al., 2013; Martini et al., 2019). B cells could produce proinflammatory cytokines including TNF-α, lymphotoxin, IL-1, and IL-6 after acquiring cytokine secretion capability. These cytokines were reported to attenuate left ventricular function and promote cardiac remodeling (Vazquez et al., 2015). Activated B cells could also directly induce cardiomyocyte damage through complement-mediated cytotoxicity (Cordero-Reyes et al., 2013). After AMI, B cells could produce CCL7 to chemoattract Ly6C^high^ monocyte to local heart, resulting in tissue damage and myocardial function deterioration (Zouggari et al., 2013). Besides, B cells produced various antibodies, which might precede disease manifestation. Several antibodies against proteins in the heart, including β1 adrenergic receptor, M2 receptor, myosin heavy-chain α and β, troponin I, Na-K-ATPase, and Kv channel, were reported to be elevated in DCM patients (Kaya et al., 2012). Antibodies deposited in the myocardium could exert direct injury and contribute to cardiac electrical instability (Cordero-Reyes et al., 2013). Serum levels of anti-heart autoantibodies were proven to be negatively associated with inotropic effects and could independently predict 5-year prognosis (Caforio et al., 2007; Kaya et al., 2012). In angiotensin II-induced HF mice model, B-cell depletion by anti-CD22 antibody resulted in reduced cardiomyocyte apoptosis, proinflammatory cytokines levels, and immunoglobulin G deposition in the myocardium; alleviated cardiac hypertrophy; and preserved left ventricular function (Cordero-Reyes et al., 2016). Therapies that depleted B cells with rituximab (CD20-specific antibody) diminished myocardial injury and improved cardiac function in patients with inflammatory DCM (Tschope et al., 2019) or AMI (phase I/II study) (Zouggari et al., 2013). Therefore, B-cell-targeted therapy is an appealing option in HF treatment.

## Extracardiac Organs

**FIGURE 1:**
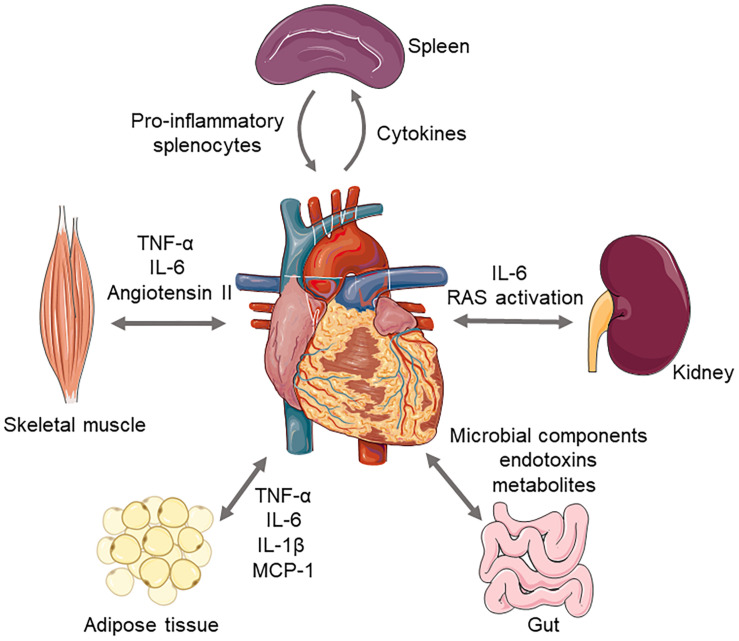
Interaction between heart and extracardiac organs. Heart failure and inflammatory responses are mutually reinforced with each other. Upon injury, splenic monocytes could mobilize and accumulate in myocardium to induce cardiac injury. Stimulus such as elevated circulating inflammatory cytokines, adipokines, and peripheral organs hypoperfusion could induce renal dysfunction, gut microbiota disorder, and skeletal muscle catabolism, which further exacerbate cardiac dysfunction.

As a systemic disease of HF, inflammatory cells and cytokines would not only affect cardiac function but also contribute to multiorgan damage through various mechanisms (Figure 1). Conversely, remote organ-related inflammatory responses would further deteriorate cardiac function.

### Cardiosplenic Axis

Clinical study that using ^18^F-fluorodeoxyglucose PET to measure the glucose metabolic rate of patients after MI revealed increased metabolic activity (reflects enhanced inflammatory cell activity) in the spleen (Wollenweber et al., 2014). Thus, remote organs might also participate in the inflammatory process of HF. Spleen harbors a large reservoir of undifferentiated monocytes. Upon injury, splenic monocytes could increase motility and accumulate in damaged tissues to regulate inflammation and promote healing process (Swirski et al., 2009). After AMI, a unique spatiotemporal pattern of a marked depletion of splenic monocytes that coincided with the accumulation of myocardial monocytes supported the hypothesis of cardiosplenic axis (van der Laan et al., 2014). HF mice underwent splenectomy showed attenuated monocyte-derived tissue macrophages and DC infiltration and reversed cardiac remodeling, whereas adoptive transfer of splenic monocytes from HF mice could induce left ventricular dysfunction and fibrosis in recipient mice (Ismahil et al., 2014). Therefore, cardiosplenic axis might play an important role in the pathogenesis of HF.

### Cardiorenal Interaction

End-stage renal disease and severe cardiovascular dysfunction are closely related to each other, which is termed as cardiorenal syndrome (CRS) (Bock and Gottlieb, 2010). Inflammatory responses, especially IL-6-related pathway, are thought to be a crucial driver of CRS in HF patients. A research containing 98 HF patients found that increased plasma IL-6 concentration was correlated with higher mortality risk, whereas elevated urine IL-6 level (quantify inflammation at the level of renal tissue) was independently associated with renal dysfunction (Hanberg et al., 2018). Previous studies demonstrated that IL-6 could impair pressure natriuresis and exacerbate renal function through activating renal epithelial sodium (ENaC) (Li K. et al., 2010) and promoting the expression of fibrotic and endothelin-1 gene (Zhang et al., 2012). Apart from the cytokine-induced renal damage, the activation of renal-angiotensin system plays a vital role in CRS. The elevated angiotensin II stimulated cardiomyocytes to release proinflammatory cytokines, including TNF-α and IL-1, which are involved in the complex mechanisms of HF (Kalra et al., 2002; Ruiz-Ortega et al., 2002).

### Gut Microbiota

Cumulative evidence has implied the significance of intestinal microbiota in various diseases including HF. The reduced cardiac output leads to intestinal ischemia, edema, and increased gut permeability, making it possible for the entry of bacteria, endotoxins, and metabolites into the bloodstream. Edematous HF patients had higher blood concentrations of lipopolysaccharide (Sandek et al., 2012). Meanwhile, higher endotoxin levels in hepatic veins than left ventricle during acute HF suggested the translocation of microbial components or endotoxins from the bowel into the circulating blood (Peschel et al., 2003). Translocation of lipopolysaccharide activated the inflammatory pathways, promoted the expression of cytokines, and contributed to HF progression (Verbrugge et al., 2013; Liu et al., 2015). Thus, probiotics and antibiotics such as rifaximin could be used in HF patients to attenuate systemic inflammation and restore metabolic homeostasis through gut microbiota modulation.

### Cardioadipose Tissue Crosstalk

The relationship between HF and obesity has long been recognized (Abel et al., 2008). Obesity is an independent risk factor for HF, especially in HFpEF. But some studies indicated that HF patients with higher body mass index and waist circumference had better prognosis than lean patients. This phenomenon has been defined as “obesity paradox” (Lavie et al., 2016; Carbone et al., 2017). Although the detailed mechanism behind this discrepancy is not clear, inflammation might be involved (Karason and Jamaly, 2020). It is widely accepted that obesity could promote systemic inflammation (Berg and Scherer, 2005; Ghigliotti et al., 2014). In obesity, visceral fat, as well as epicardial and pericardial fat, enhanced the expression of various proinflammatory cytokines, including TNF-α, IL-6, IL-1β, and monocyte chemoattractant protein 1, whereas it reduced the expression of anti-inflammatory cytokines, such as IL-10 and adiponectin (Jahng et al., 2016). The chronic systemic inflammation in obesity further promoted the accumulation of epicardial fat and adversely damaged the biology of epicardial fat toward a proinflammatory phenotype (Hirata et al., 2011; Wernstedt Asterholm et al., 2014). The proinflammatory adipocytokines, gaseous messengers, and lipids secreted by epicardial adipose tissue could affect cardiomyocytes and extracellular matrix through a paracrine manner (Patel et al., 2017). Thus, epicardial fat could serve as a transducer that mediated the influence of systemic inflammation on adjacent myocardium (Packer, 2018). This might partially explain the cardiac sterile inflammation in obese people. On the other hand, in HF patients, damaged cardiomyocytes could release proinflammatory cytokines, such as IL-6 and TNF-α, which could trigger lipolysis of epicardial adipose tissue, leading to cardiac cachexia and worsening outcome (Oikonomou and Antoniades, 2019). Strategies that reduced the quantity of epicardial adipose tissue, such as high doses of statins (Abe et al., 2008; Alexopoulos et al., 2013; Cho et al., 2015; Yamada et al., 2017), metformin (Jonker et al., 2010; Cameron et al., 2016), mineralocorticoid receptor antagonists (Guo et al., 2008; Anand et al., 2017; Olivier et al., 2017), sodium–glucose cotransporter 2 inhibitors (Habibi et al., 2017; Lee et al., 2017), as well as low-calorie diets and physical exercise (Kim et al., 1985; Kelly et al., 2014), could reduce systemic inflammation, prevent, or treat HFpEF.

### Heart and Skeletal Muscle Crosstalk

Heart failure patients are frequently accompanied with skeletal muscle wasting, which is generally not associated with body weight loss but mainly due to the imbalance of the muscle protein synthesis and degradation (von Haehling et al., 2013; Ebner et al., 2014). The elevated circulating cytokines such as TNF-α and IL-6 in HF patients could induce muscle protein loss by activating nuclear factor κB pathway (Li et al., 1998; Lavine and Sierra, 2017) and lead to skeletal muscle apoptosis through promoting sphingosine production (Dalla Libera et al., 2001). Besides, activated angiotensin II in HF patients was involved in the metabolism of skeletal muscle (Delafontaine and Akao, 2006; Sukhanov et al., 2011). In HF patients, damaged or dying myocytes could release various danger-associated molecular patterns and myokines, such as myostatin, IL-8, IL-15, and osteonectin, into plasma, which contribute to HF-related myopathy (Chan et al., 2012; Berezin et al., 2021).

## Conclusion

Immune activation possesses a vital role in the progression of HF. However, anti-inflammatory clinical trials showed limited success. The diverse clinical etiologies and the intrinsic complexity of inflammatory responses may partially explain these unsatisfied results. Further insights and clinical trials about inflammation in specific etiologies and stages of HF are needed (Table 3). Furthermore, stratifying the HF patients into particular subpopulations according to their inflammatory conditions may maximize the effects of anti-inflammatory therapy.

**TABLE 3:** Ongoing clinical trials targeting inflammation.

Drug	Trial identifier	Disease	Primary endpoint	Duration of therapy	Phase and status	Sponsor
Anakinra (interleukin 1 blockade)	NCT03797001	Heart failure, systolic, inflammation	Changes in peak VO_2_ at earlier endpoints	24 Weeks	Phase 2; recruiting	Virginia Commonwealth University
Proleukin (interleukin 2)	NCT03113773	Ischemic heart disease	–	5 Days	Phase 1/2; active, not recruiting	Cambridge University Hospitals NHS Foundation Trust
Interleukin 2 (IL-2)	NCT04241601	Acute coronary syndromes	Change in vascular inflammation	5 Days	Phase 2; recruiting	Cambridge University Hospitals NHS Foundation Trust
Colchicine (anti-inflammatory)	NCT04857931	Heart failure, inflammation	Change in hs-CRP (C-reactive protein)	–	Phase 3; not yet recruiting	Montreal Heart Institute
Colchicine (anti-inflammatory)	NCT04420624	Myocardial infarction, acute	Percentage of myocardial denervation	1 Month	Phase 2/3; recruiting	University Hospital, Montpellier

## Author Contributions

HL and CC conceived and wrote the manuscript. DW supervised and wrote the manuscript. All authors contributed to the article and approved the submitted version.

## Conflict of Interest

The authors declare that the research was conducted in the absence of any commercial or financial relationships that could be construed as a potential conflict of interest.
